# Quercetin and Resveratrol Differentially Decrease Expression of the High-Affinity IgE Receptor (FcεRI) by Human and Mouse Mast Cells

**DOI:** 10.3390/molecules27196704

**Published:** 2022-10-08

**Authors:** Syed Benazir Alam, Ashley Wagner, Steven Willows, Marianna Kulka

**Affiliations:** 1 Nanotechnology Research Center, National Research Council, Edmonton, AB T6G 2M9, Canada; 2 Medical Microbiology and Immunology, University of Alberta, Edmonton, AB T6G 2E1, Canada

**Keywords:** mast cells, IgE, FcεRI receptor, quercetin, resveratrol, degranulation, cytokine

## Abstract

Mast cells (MC) synthesize and store proinflammatory mediators and are centrally important in atopic diseases such as asthma and atopic dermatitis. Quercetin a and resveratrol are plant derived polyphenolic compounds with anti-inflammatory properties that inhibit MC degranulation and mediator release. However, the underlying mechanism of these inhibitory effects on MC is poorly understood and it is unclear whether this is a general effect on all MC phenotypes. We have characterized and compared the effects of quercetin with resveratrol on human (LAD2) and mouse (MC/9 and BMMC) MC mediator release, receptor expression and FcεRI signaling to better understand the mechanisms involved in quercetin and resveratrol-mediated inhibition of MC activation. Quercetin significantly decreased the expression of FcεRI by BMMC and MC/9, although the effects on MC/9 were associated with a significant reduction in cell viability. Quercetin also inhibited antigen-stimulated TNF release by BMMC. Although neither quercetin nor resveratrol significantly altered antigen-stimulated BMMC degranulation or downstream signaling events such as phosphorylation of spleen tyrosine kinase (SYK) or extracellular signal-regulated kinase 1/2 (ERK), resveratrol inhibited ERK phosphorylation and FcεRI- stimulated degranulation in LAD2. Our data suggests that quercetin and resveratrol inhibit human and mouse MC differentially and that these effects are associated with modification of FcεRI expression, signaling (phosphorylation of SYK and ERK) and mediator release.

## 1. Introduction

Quercetin (3,3′,4′,5,7-pentahydroxyflavone) is a naturally occurring flavonoid that is abundant in vegetables and fruits such as onions, garlic, ginger, broccoli, peppers, buckwheat, apples, grapes, berry crops, citrus fruits including some herbs, tea and wine [1,2]. Quercetin has antioxidant, antiviral, antibacterial, anti-inflammatory and anticarcinogenic properties [3,4,5,6,7]. The multifunctional role of quercetin has been attributed to the inhibition of key signaling enzymes such as protein kinase C, phosphatidylinositol 3-kinase and tyrosine kinase some of which are involved in the regulation of cell proliferation, angiogenesis, apoptosis and inhibition of DNA damage [8]. Due to its anti-inflammatory and anti-allergic properties quercetin is a possible treatment for respiratory and food allergies as well as contact dermatitis and photosensitivity [9,10,11,12]. In animal models, quercetin suppresses anaphylactic responses in sensitized rats [13] and inhalation of quercetin inhibits asthmatic inflammation in guinea pigs and rats [14]. In vitro, quercetin inhibits human and rodent mast cell (MC) mediator release, although the data is often scattered and is reported from different cell types as well as evaluates only a small spectrum of MC functions [15,16,17,18].

MC are myeloid-derived immune cells that are present in connective tissues and mucosal surfaces and play an important role in inflammatory and homeostatic responses [19]. They originate from stem cells in the yolk sac during embryogenesis and postnatally from pluripotent progenitor stem cells in the bone marrow that differentiate and mature under the influence of the c-kit (KIT) receptor ligand (stem cell factor; SCF) and other growth factors in the microenvironment of the tissue where they reside [20]. MC mediate allergic inflammatory responses [21], and they are activated when allergen binds their surface high-affinity IgE receptors (FcεRI) [22]. FcεRI is a tetramer and when clustered by IgE/allergen binding, it initiates complex intracellular signaling cascades that result in several MC functions, including degranulation, cytokine/chemokine production, reactive oxygen species generation (ROS), arachidonic acid metabolite release, chemotaxis and adhesion [23,24]. Crosslinking of IgE bound-FcεRI by multivalent allergens causes a rapid release of preformed mediators that are contained within granules, such as typtase, chymase, histamine, proteoglycans, metaloproteinases and β-hexosaminidase. This rapid response is called degranulation and occurs within seconds after activation. MC can also be activated via ligation of the mas-related G protein-coupled receptor (MRGPRX2) by agonists such as compound 48/80 and substance P, adenosine receptors [25,26] or through receptor-independent activation of intracellular signaling with phorbol 12-myristate 13-acetate (PMA, or 12-O-tetradecanoylphorbol 13-acetate, TPA) and ionophore [27,28].

Quercetin decreases the release of histamine in vitro [29,30] although the precise mechanisms of this inhibition are poorly understood. Previous work from our lab has shown that resveratrol (3,5,4′-tryhydroxytrans-stilbene), a polyphenol abundant in peanuts, red wine and the skin of grapes, inhibits degranulation of human Laboratory of Allergic Diseases (LAD)2 MC that are activated by substance P (SP), IgE crosslinking and compound 48/80 [31]. Resveratrol attenuates SP-induced TNF and MCP-1 production and inhibits IgE-mediated release of cysteinyl leukotrienes. In addition, resveratrol inhibits the expression of the high-affinity IgE receptor, FcεRI on LAD2 cells [31]. Resveratrol prevents increase in MC in both ovalbumin-induced allergic enteritis as well as experimental cholitis in IL-10^−/−^ mice [32] and inhibits IgE-mediated rat basophilic MC degranulation and passive cuteneous anaphylaxis in mice [33]. It also inhibits human intestinal MC activation by inhibiting the phosphorylation of mitochondrial and nuclear extracellular signal-regulated kinase 1/2 (ERK) and signal transducer and activator of transcription (STAT) 3 [34].

Quercetin and resveratrol are structurally different, but, they both are polyphenolic compounds that are ubiquitously present in vegetables, fruits and beverages and have similar anti-allergic inflammatory properties that are gaining interest as a nutraceutical [1,35,36,37], although the underlying molecular mechanism of MC inhibition is unexplored. In the present study, we evaluated and compared the effects of quercetin and resveratrol on FcεRI expression, SYK/ERK signaling as well as mediator release in different types of MC models (from mice and humans). Our results show that quercetin and resveratrol differentially decrease expression of FcεRI and that this effect is species specific.

## 2. Results

### 2.1. Effects of Quercetin and Resveratrol on FcεRI Expression and TNF Release by MC/9

#### 2.1.1. Quercetin Inhibits the Expression of FcεRI by MC/9

**Figure 1:**
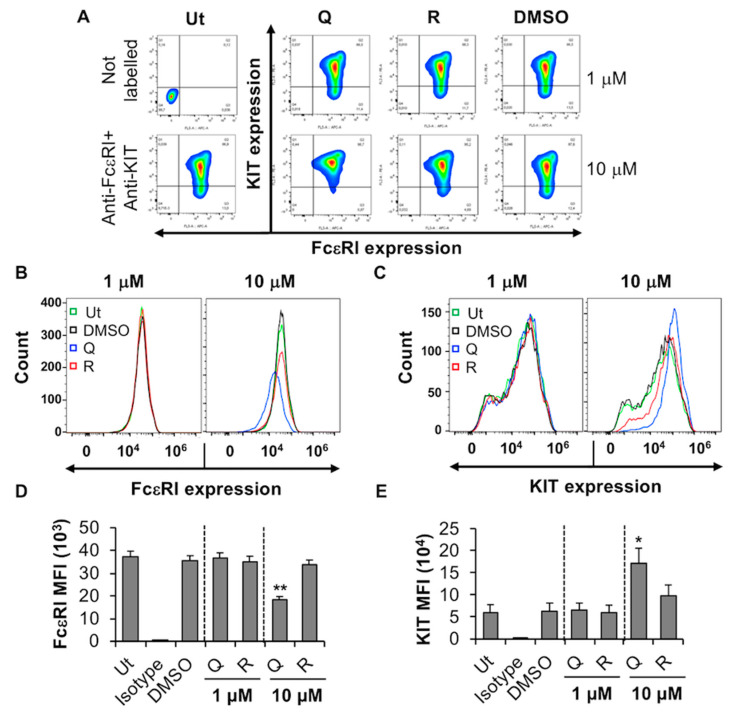
Effects of resveratrol and quercetin on FcεRI and KIT expression by MC/9. (**A**) MC/9 were treated with 1 or 10 μM quercetin (Q), resveratrol (R) or DMSO for 24 h followed by flow cytometry analysis. Ut represents untreated MC/9. FcεRI versus KIT expression contour plots were generated relative to the untreated unstained sample shown on the left-hand side panel. (**B**,**C**) Histogram overlay showing FcεRI and KIT expression by MC/9 treated with 1 or 10 µM Q, R or DMSO as in A. (**D**,**E**) Mean fluorescence intensity (MFI) of FcεRI and KIT expression by MC/9 treated as in A. n = 6. *p* values < 0.05 (*) and < 0.01 (**) are relative to DMSO control.

MC/9 is an IL-3-dependent mouse MC line that expresses high levels of FcεRI [38], but low levels of the stem cell factor receptor, KIT/CD117. FcεRI is the high-affinity IgE receptor that is expressed on mature connective tissue and mucosal MC and serves as an important activation signal during allergic inflammation [39,40,41]. The effect of quercetin and resveratrol on FcεRI and KIT expression by MC/9 was determined by flow cytometry analysis. Quercetin at a concentration of 1 µM had no significant effect on FcεRI expression by MC/9. However, when compared to the DMSO control, 10 µM quercetin decreased the expression of FcεRI as indicated by a decrease in fluorescence of the total cell popullation (Figure 1A, compare 10 µM “Q” with DMSO) and a decrease in overall average mean fluorescence intensity (MFI) of the gated cell population (Figure 1D). Ten µM quercetin caused an increase in the expression of KIT (Figure 1E) as indicated by a shift of overal fluorescence of the cell population towards the right (Figure 1C) and an increase in MFI (Figure 1E). Viability analysis using trypan blue exclusion assays, indicated that quercetin significantly decreased MC/9 viability at 10 µM (Supplementary Figure S1(Ai)). Moreover, a dose response cytotoxicity assay (1–10 µM quercetin) showed that quercetin is toxic to MC/9 starting at 2–4 µM (Supplementary Figure S1(Aii)). Hence, the decrease in FcεRI and increase in KIT expression after treatment with 10 µM quercetin may be due to a loss in cell viability. Resveratrol did not have any cytotoxic effects up to 30 µM (Supplementary Figure S1(Ai)), and did not change FcεRI and KIT expression by MC/9 (Figure 1B,C; compare “R” with DMSO). Altogether these results suggest that quercetin-induced reduction in FcεRI expression by MC/9 may be due to a decrease in cell viability.

#### 2.1.2. Quercetin Inhibits TNF Release from MC/9

**Figure 2:**
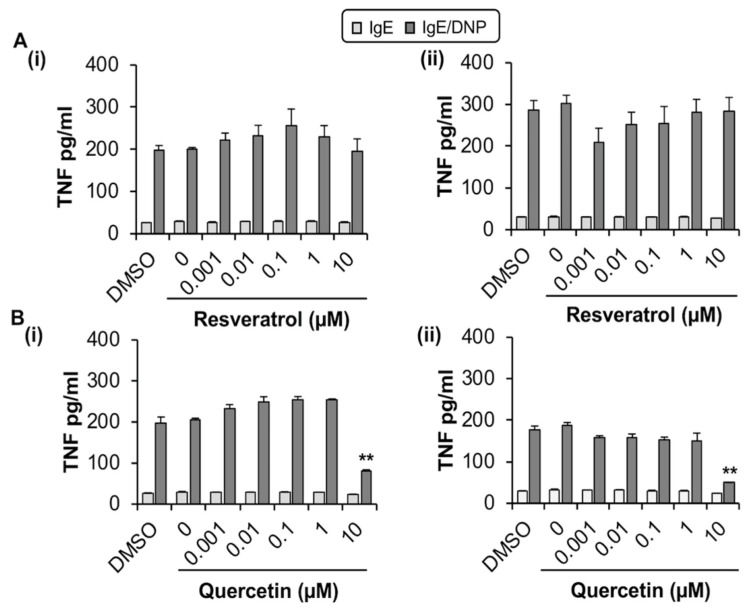
Effects of resveratrol (**A**) and quercetin (**B**) on TNF release by MC/9. (**A**,**B**) MC/9 (1 × 10^6^ cells/mL) were sensitized with IgE and treated with resveratrol or quercetin (0–10 μM) and the DMSO control for (i) 1 h or (ii) 24 h followed by stimulation with 100 ng/mL DNP. n = 3. *p* value < 0.01 (**) is relative to DMSO control.

We next evaluated whether quercetin and resveratrol modified the release of TNF from antigen-activated MC/9 by ELISA. Optimization experiments indicated that MC/9 release the pro-inflammatory cytokine TNF after sensitization with IgE and stimulation with antigen through crosslinking of surface FcεRI (Figure S1B,C). Quercetin reduced TNF production by MC/9 by 59% (1 h; Figure 2(Bi)) and 99% (24 h; Figure 2(Bii)) when compared to DMSO control but only at 10 µM, which is significantly cytotoxic to MC/9. However, at non-cytotoxic concentrations of quercetin (1 µM), there were no changes in FcεRI expression. In contrast, resveratrol, which was not cytotoxic up to 30 µM (Supplementary Figure S1(Ai)), did not affect TNF release from activated MC/9 (Figure 2(Ai,ii)), and did not change FcεRI expression (Figure 1A,B,D). Hence, due to the lack of change in FcεRI expression by non-cytotoxic concentrations of quercetin and resveratrol, MC/9 were deemed to be an inadequate model for further study of phytochemical effects on MC FcεRI expression and function.

### 2.2. Effect of Quercetin and Resveratrol on FcεRI Expression, FcεRI-Mediated Signaling, Degranulation and TNF Release by BMMC

#### 2.2.1. Quercetin and Resveratrol Inhibit the Expression of FcεRI by BMMC

BMMC are mouse mast cells that have been differentiated from bone marrow progenitor cells using IL-3. After 4 weeks in culture, the resulting BMMC are granulated, express functional FcεRI receptors, respond to IgE/antigen stimulation and produce a plethora of cytokines and chemokines [42]. Viability analysis indicated that quercetin had no significant effect on BMMC viability at 10 µM, but increased slightly between 30 and 100 µM (Supplementary Figure S2). This data supports the observation made by Krajewski et al. that 100 µM quercetin significantly decreased BMMC viability after 24 h [43]. By contrast, resveratrol had no significant effect on BMMC viability at similar concentrations.

**Figure 3:**
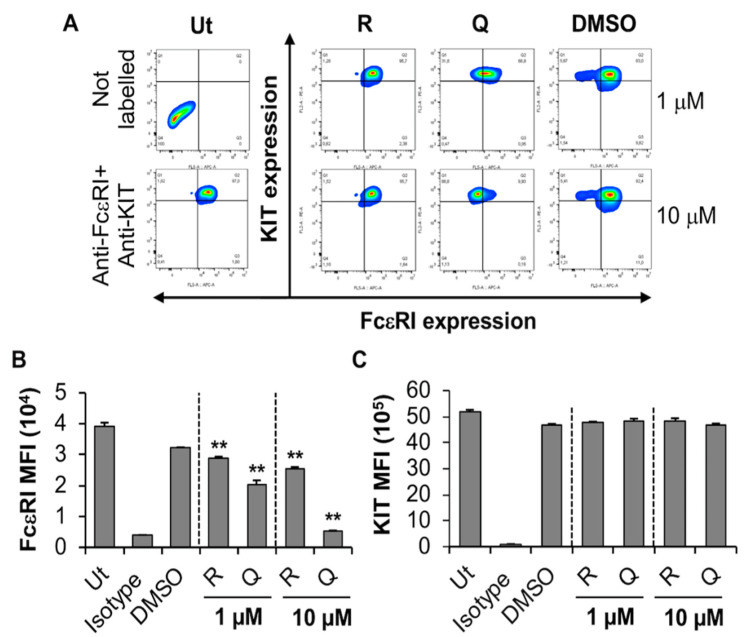
Effects of resveratrol and quercetin on FcεRI and KIT expression by BMMC. (**A**) BMMC were treated with 1 or 10 μM quercetin (Q), resveratrol (R) or DMSO for 24 h followed by flow cytometry analysis. Ut represents untreated BMMC. FcεRI versus KIT expression contour plots were generated relative to the untreated unstained sample shown on the left-hand side panel. (**B**,**C**) Mean fluorescence intensity (MFI) of FcεRI and KIT expression by BMMC treated as in A. n = 3. *p* values < 0.01 (**) are relative to DMSO control.

The effect of quercetin and resveratrol on BMMC expression of FcεRI was evaluated using flow cytometry and biocompatible concentrations (1 and 10 µM) of the phytochemicals (Figure 3). Resveratrol and quercetin significantly inhibited the expression of FcεRI in a concentration-dependent manner (Figure 3A, compare shift in contour plots of “Q” and “R” with DMSO; Figure 3B, compare dose-dependent reduction in FcεRI MFI of “Q” and “R” with DMSO) while having no effect on the expression of the KIT receptor (Figure 3C). Although DMSO appears to cause some shift in FcεRI expression in a subset of BMMC (Figure 3A, compare a small subpopulation in top-left hand side quadrant in DMSO treated sample with that of untreated, Ut), the overall shift in FcεRI expression in the entire population is only observed when BMMC is treated with resveratrol and quercetin. This effect is especially significant when BMMC are treated with 10 µM quercetin (Figure 3A,B, compare the shift of total cell population from top right-hand side quadrant in DMSO control to top left-hand side quadrant in 10 µM “Q” treated samples). Of the two compounds that were tested, quercetin showed the most potent effect on the expression of FcεRI, where a 38% to 83% reduction was observed with 1 and 10 µM, respectively (Figure 1B). In contrast, none of the compounds modified KIT expression on BMMC (Figure 3C). This suggests that quercetin and resveratrol significantly inhibit the expression of FcεRI without affecting KIT expression in primary mouse MC, BMMC, with quercetin and resveratrol having the most prominent effects at 10 µM.

#### 2.2.2. Quercetin Inhibits the Release of TNF from BMMC but Has No Measurable Effect on Phosphorylation of SYK and ERK or Degranulation

**Figure 4:**
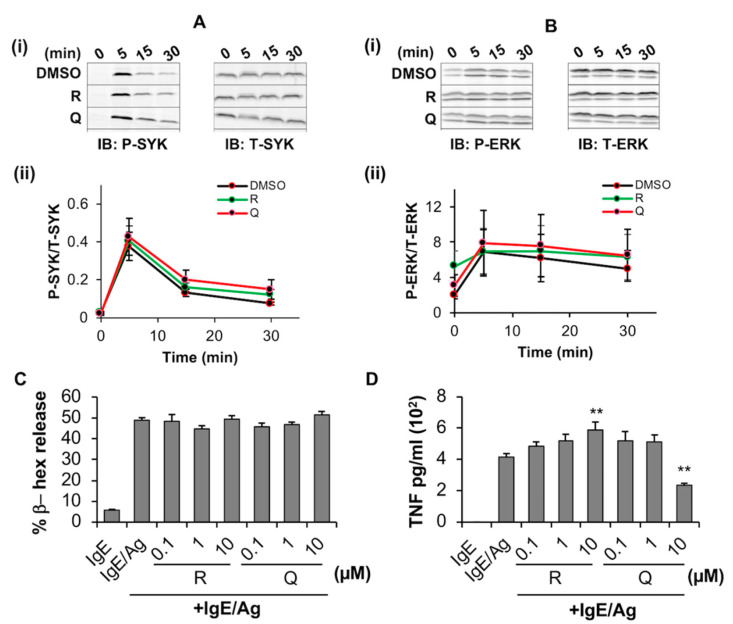
Effects of resveratrol and quercetin on SYK/ERK phosphorylation and degranulation by BMMC. (**A**,**B**) BMMC were treated with 10 μM resveratrol (R), quercetin (Q), or an equal volume of DMSO or media and incubated for 24 h while simultaneously exposed to 100 ng/mL of anti-DNP IgE. Activation of the IgE receptor was then induced by exposure to 100 ng/mL DNP-BSA for indicated times before lysis. Lysates were then probed via Western blot with antibodies towards phosphorylated (P) and (T) total SYK (A-i) and ERK1/2 (ERK, B-i). Densitometric analysis of the ratio between “P” and “T” stains are shown for SYK (A-ii) and ERK (B-ii) (n = 3). (**C**) BMMC were sensitized with anti-DNP IgE overnight and simultaneously treated with 0.1, 1 and 10 µM resveratrol (R) or quercetin (Q) for 24 h followed by stimulation with DNP-BSA and measuring the % release of β-hex (**D**). BMMC were sensitized with anti-DNP IgE overnight and treated with 0.1, 1 and 10 µM resveratrol (R) or quercetin (Q) for 24 h followed by stimulation with DNP-BSA and measuring the % release of TNF. n = 3. *p* value < 0.01 (**) are relative to IgE/Ag.

We next evaluated the effects of these phytochemicals on FcεRI -mediated signaling, BMMC degranulation and cytokine release. BMMCs were treated with the phytochemicals and simultaneously sensitized with anti-DNP IgE for 24 h. IgE receptor was activated by exposure to the antigen DNP-BSA for 5–30 min followed by lysis and Western blot analysis to evaluate the effect of phytochemicals on signaling events particularly phosphorylation of the adaptor proteins SYK and ERK post FcεRI activation. Immunoblotting (Figure 4(Ai,Bi)), compare phospho (P)-SYK and P-ERK blots of “Q” and “R” at each time point to total (T)-SYK and T-ERK blots at the corresponding time points of DMSO control] and densitometric analysis (Figure 4(Aii,Bii)), compare P-SYK/ERK to T-SYK/ERK line graphs of “Q” and “R” to that of DMSO] indicated that resveratrol and quercetin had no effects on antigen-induced phosphorylation of SYK and ERK after at least 5 min following activation when compared to DMSO control.

Resveratrol and quercetin also had no significant effect on antigen (Ag)-activated BMMC degranulation (Figure 4C) as indicated by no changes in the release of the β-hexosaminidase (β-hex) enzyme. However, ELISA showed that 10 µM quercetin significantly inhibited the release of TNF from antigen-activated BMMC by 44% (Figure 4D) when compared to BMMCs that were activated with IgE/antigen. Interestingly, resveratrol potentiated the release of TNF in a concentration-dependent manner up to 10 µM suggesting that resveratrol and quercetin have opposite effects on TNF production by IgE/Ag-activated BMMC, at least under these culture conditions.

### 2.3. Effect of Quercetin and Resveratrol on FcεRI Expression, FcεRI-Mediated Signaling and Degranulation by LAD2

#### 2.3.1. Quercetin and Resveratrol Inhibit the Expression of KIT While Moderately Affecting FcεRI Expression by the Human MC Line, LAD2

**Figure 5:**
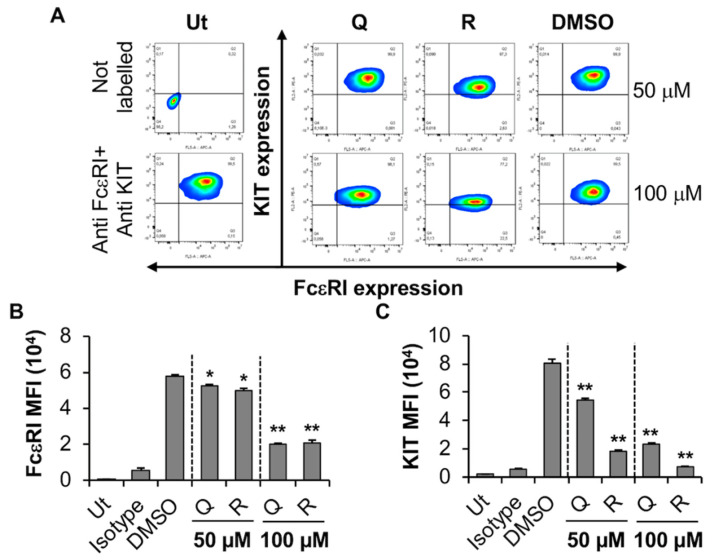
Effects of resveratrol and quercetin on FcεRI and KIT expression by LAD2. (**A**) LAD2 were treated with 50 or 100 μM quercetin (Q), resveratrol (R) or DMSO for 24 h followed by flow cytometry analysis. Ut represents untreated LAD2. FcεRI versus KIT expression contour plots were generated relative to the untreated unstained sample shown on the left-hand side panel. (**B**,**C**) Mean fluorescence intensity (MFI) of FcεRI and KIT expression by LAD2 treated as in A. n = 3. *p* values < 0.05 (*) and <0.01 (**) are relative to DMSO control.

We then evaluated the effect of quercetin and resveratrol on the human MC line LAD2. LAD2 are reasonably granular, express a functional FcεRI receptor, are dependent upon SCF for proliferation, and express both tryptase and chymase. Viability analysis indicated that even the highest concentrations of compounds (100 µM) had no significant effect on LAD2 viability (Supplementary Figure S3A). Flow cytometry analysis showed that resveratrol and quercetin significantly inhibited the expression of KIT receptor by LAD2 in a concentration-dependent manner as indicated by a shift in contour plots towards the bottom right-hand side quadrant (Q3) from the top right-hand side quadrant (Q2) (Figure 5A) as well as a significant reduction in MFI (Figure 5C) when compared to DMSO control. In contrast to BMMC, both the phytochemicals significantly decreased FcεRI expression by LAD2 cells (Figure 5A,B). Hence, these results suggest that the KIT receptor is much more sensitive to the effects of quercetin and resveratrol than FcεRI in LAD2 cells. We hypothesized that perhaps the changes in FcεRI expression may occur through changes in gene transcription. Thus, LAD2 were treated with 100 µM quercetin or resveratrol and the genes for the α, β and γ subunits of FcεRI were measured using qRT-PCR. Our results showed that phytochemicals did not alter the expression of FcεRI subunit genes (Supplementary Figure S3B). This result suggests that quercetin and resveratrol-mediated reduction in FcεRI expression in LAD2 cells does not occur by changes in gene transcription of the receptor subunits.

#### 2.3.2. Resveratrol Inhibits FcεRI-Dependent Signaling and Degranulation in LAD2

**Figure 6:**
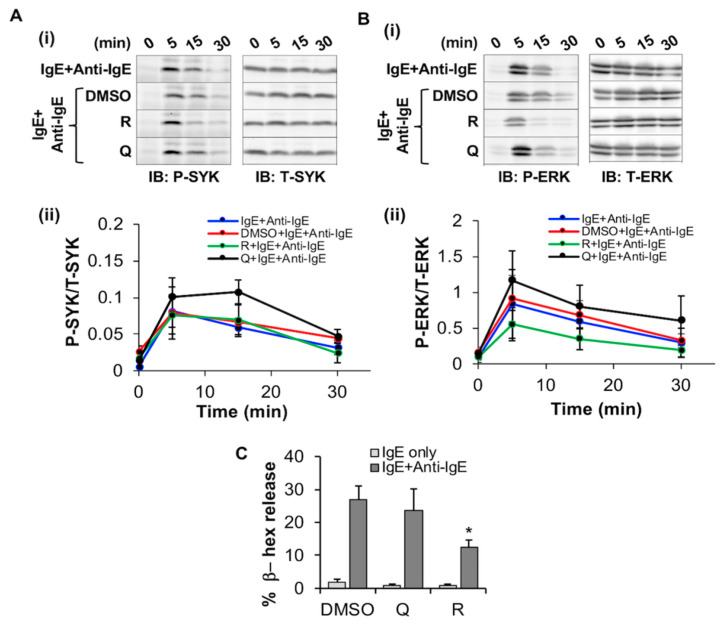
Effects of resveratrol and quercetin on SYK and ERK phosphorylation and degranulation in LAD2. (**A**,**B**) LAD2 cells were treated with 50 μM resveratrol (R), quercetin (Q) and an equal volume of DMSO or media and incubated for 24 h while simultaneously exposed to 100 ng/mL of human IgE. Activation of the IgE receptor was then induced by exposure to 10 µg/mL anti-IgE for indicated times before lysis. Lysates were then probed via Western blot with antibodies towards phosphorylated (P) and total (T) SYK (A-i) and ERK1/2 (ERK, B-i). Densitometric analysis of the ratio between “P” and “T” stains are shown for SYK (A-ii) and ERK (B-ii). n = 3. (**C**) LAD2 cells were treated with 50 μM resveratrol (R), quercetin (Q) and an equal volume of DMSO or media and incubated for 24 h followed by sensitization with IgE for 6 h and measuring degranulation as % β-hex release, following 30 min stimulation with 10 µg/mL IgE. n = 5. Statistically significant data with *p* value < 0.05 are represented as *.

Since FcεRI expression was significantly decreased by quercetin and resveratrol, we hypothesized that this may alter downstream signaling via SYK and ERK phosphorylation. We thus assessed the effect of these phytochemicals on FcεRI -mediated signaling by measuring phosphorylation of SYK and ERK relative to total SYK and ERK using Western blot analysis. LAD2 were simultaneously treated with the phytochemicals and sensitized with human IgE followed by activation using anti-IgE for 5–30 min. When compared to the DMSO control, the phytochemicals had no significant effect on SYK phosphorylation (Figure 6A) relative to total SYK). However, resveratrol inhibited ERK phosphorylation relative to total ERK by 15 min post anti-IgE treatment when compared to DMSO control (Figure 6B) as determined by both immunoblotting (Figure 6(Bi), compare P-ERK blot of “R+IgE+Anti-IgE” at 15 and 30 min to that of “DMSO+IgE+Ant-IgE” control) and densitometric analysis (Figure 6(Bii), compare “R+IgE+Anti-IgE” line graph to that of “DMSO+IgE+Anti-IgE”). In contrast, quercetin had no effects on ERK phosphorylation at these time points, and densitometric analysis showed no statistically significant differences between the treatment groups.

Since SYK, but not ERK, phosphorylation is associated with the downstream process of degranulation, we determined whether these compounds had any effect on degranulation of LAD2. Resveratrol inhibited IgE/anti-IgE-stimulated LAD2 degranulation (Figure 6C) as determined by a significant decrease in the percentage release of β-hex enzyme. In contrast, quercetin, had no significant effects on LAD2 degranulation. Altogether, these results suggest that resveratrol reduces ERK phosphorylation as well as degranulation from FcεRI-activated LAD2 human MC.

## 3. Discussion

Plant derived medicinal compounds or phytochemicals have become increasingly popular due to their fewer side effects, low cost and natural origin. Quercetin and resveratrol are one of the most common polyphenolic compounds consumed by humans through food [44,45]. Although the effects of quercetin and resveratrol on MC activation have been previously reported [35,36], it is unclear how these effects manifest across different MC phenotypes from different species. Furthermore, the mechanisms of quercetin and resveratrol effects on MC functions are poorly understood. Our study makes a novel observation that quercetin and resveratrol inhibit FcεRI expression in BMMC in a concentration-dependent manner with a subsequent decrease in TNF release, while having no effect on SYK/ERK phosphorylation and degranulation. To our knowledge, this is the first comprehensive study to compare the effect of quercetin and resveratrol in different types of MC (rodent and human) and to analyze functional responses (degranulation, cytokine release and FcεRI/KIT expression). We also studied the effects of these compounds on MC/9 (that do not possess mature granules containing pre-stored mediators like histamine unlike BMMC) and BMMC as two different maturity models of mast cells. We observed that MC/9 is not an ideal model system to study the effect of quercetin in MC due to underlying cytotoxicity and the fact that non-cytotoxic concentration of phytochemicals was unable to modify FcεRI expression.

Quercetin exhibits strong anti-inflammatory properties. When used in combination with other flavonoids, it has been used as a treatment of acute and chronic inflammatory conditions such as asthma [46]. Quercetin regulates Th1/Th2 balance in mouse models of asthma [47] as well as human peripheral blood derived CD4^+^ T cells [48]. Quercetin plays an important role in the treatment of allergic rhinitis (AR) and atopic dermatitis (AD) [12]. Resveratrol has also been studied for its anti-allergic inflammatory properties [31,33,49,50,51], however a thorough comparative analysis of the effects of quercetin and resveratrol across different species and MC types as well as the underlying molecular mechanism associated with the inhibition of MC activation still remains an area of investigation.

MC play a prominent role in the disease etiology of inflammatory diseases such as asthma, allergy, AR, AD. MCs are mainly found at the host-environment interface such as the skin, lung or the gastrointestinal tract and these areas are regularly challenged by allergens or pathogens. MCs can identify and respond to a number of endogenous (cytokines, chemokines, IgG, adenosine, anaphylatoxins, neuropeptides such as substance P) and exogenous (some contents of insect venom, polycationic molecules such as compound 48/80, pathogen associated molecular patterns, many drugs) stimuli [20,50]. MC contain and release several pro-inflammatory compounds such as histamine, tryptase, arachidonic acid metabolites, cytokines and chemokines. Glycoproteins, allergens or auto-antibodies directed against the FcεRI or receptor-bound IgE, can cause MC degranulation after cross-linking and aggregation of the surface-bound receptor FcεRI. Quercetin inhibits the release of histamine and monocyte chemoattractant protein-1, a chemokine released by activated MCs to regulate the migration and infiltration of monocytes and macrophages, from HMC-1 cells [51]. Quercetin inhibits the secretion of IL-6 from IL-1-activated HMC-1 as well as reduces p38 and protein kinase C (PKC) phosphorylation, key events that are induced by the pro-inflammatory cytokine IL-1 [52]. Additionally, quercetin inhibits the transcription of histidine decarboxylase, the enzyme responsible for the generation of histamine from histidine. Quercetin negatively regulates the FcεRI signaling pathway [52] by inhibiting key signaling enzymes such as phosphatidylinositol-3-phosphate kinase (PI-3K) and components of the mitogen-activated protein kinase (MAPK) pathway [53]. While the molecular mechanisms are not fully understood, quercetin has been shown to inhibit SYK phosphorylation after FcεRI activation, but not phosphorylation of Lyn [54]. This suggests that either Lyn is directly targeted by quercetin, thus preventing downstream phosphorylation of SYK, or SYK is the target and it is autophosphorylation that is prevented. Although several studies have shown that quercetin inhibits FcεRI signaling and mediator release, the direct effect of quercetin on FcεRI expression is unexplored.

**Figure 7:**
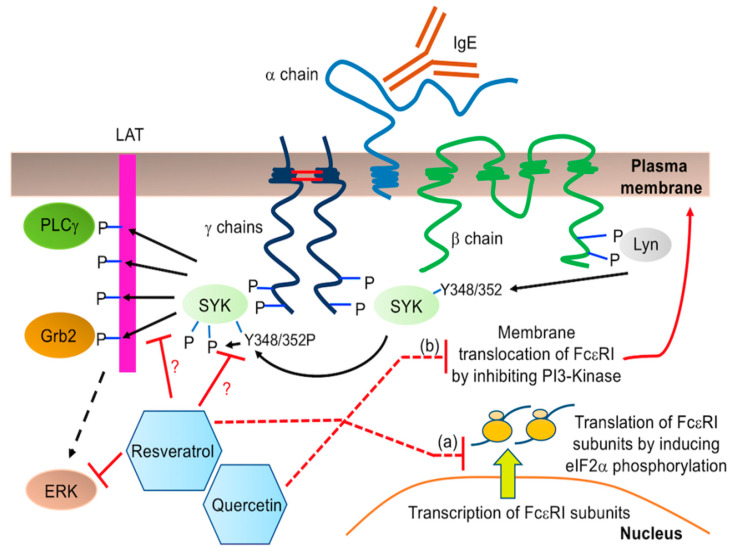
Schematic of FcεRI structure and signaling. The receptor is composed of 4 subunits: one α chain, one β chain and two γ chains joined by two disulphide bridges. When activated by IgE crosslinking, FcεRI phosphorylates and recruits adaptor proteins Lyn and SYK. Lyn phosphorylation of SYK, especially at tyrosine (Y) residues 348 and 352, and SYK’s autophosphorylation, leads to activation of SYK which then phosphorylates LAT, allowing for recruitment of PLC γ and Grb2. Resveratrol may inhibit this pathway downstream of tyrosine 348 phosphorylation, which is detected by the specific anti-phospho-SYK antibody used in this study. It may inhibit SYK autophosphorylation or phosphorylation of the downstream targets to ultimately prevent ERK phosphorylation. Some possible mechanisms by which resveratrol and quercetin might inhibit FcεRI surface expression include: (**a**) translation of FcεRI subunit mRNAs via inducing phosphorylation of eukaryotic initiation factor 2α (eIF2α) or (**b**) vesicular trafficking of FcεRI complex from endoplasmic reticulum to the plasma membrane via inhibiting phosphoinositide 3-kinase (PI3-Kinase).

We have made a novel observation in this study that phytochemicals differentially modify cell surface expression of FcεRI and KIT in human and mouse MC. Quercetin causes a drastic reduction in FcεRI expression in BMMC (Figure 3) and this correlates with a decrease in *de novo* cytokine (TNF) production (Figure 4D) which confirms earlier reports by Jeong et al. and Krajewski et al. [43,55]. The mechanism behind this change in cell surface expression of FcεRI is currently unknown. Our results show that neither quercetin nor resveratrol altered mRNA expression of any of the FcεRI subunits α, β or γ by LAD2 (Figure S3B). Hence, it is possible that phytochemicals inhibit the cell surface expression of FcεRI at the post-transcriptional level. Increased phosphorylation of eukaryotic initiation factor 2α (eIF2α) causes inhibition of global translation and quercetin induces eIF2α phosphorylation [56]. Hence, it is possible that quercetin inhibits FcεRI translation by increasing eIF2α phosphorylation. FcεRI protein complex is assembled in the endoplasmic reticulum and then translocates to the plasma membrane. Quercetin inhibits phosphoinositide 3-kinase (PI3-kinase) [57,58,59] that plays an important role in vesicular trafficking [60]. Quercetin supresses insulin-dependent translocation of glucose transporter 4 to the plasma membrane via inhibiting PI3-kinase [61]. Hence, it is possible that quercetin inhibits translocation of FcεRI from ER to the plasma membrane. Figure 7 depicts possible mechanisms by which quercetin and resveratrol could be inhibiting cell surface expression of FcεRI in MC.

SYK and ERK signaling pathways play an important role in orchestrating FcεRI- mediated MC signal transduction, leading to mediator release. Interestingly, our data suggests that quercetin and resveratrol did not alter SYK and ERK phosphorylation and degranulation by BMMC activated through FcεRI despite lower cell surface expression of FcεRI. There are several possibilities for this novel observation. First, our Western blot analysis measured phosphorylation of these proteins only after 5 min of stimulation of the FcεRI. Since membrane-proximal phosphorylation of SYK is very rapid, it is possible that the time at which these compounds modify SYK phosphorylation is earlier than 5 min. Second, it is possible that the threshold for FcεRI expression to significantly impact SYK phosphorylation has not been reached with quercetin or resveratrol and the cell is still capable of a robust SYK response despite the change in FcεRI expression. Third, in several experiments, including those looking at SYK/ERK phosphorylation and degranulation in BMMC, cells were simultaneously treated with resveratrol/quercetin and IgE. In other experiments, including those looking at FcεRI expression by flow cytometry, TNF release in BMMC and degranulation in LAD2, cells were exposed to quercetin and resveratrol before/without IgE sensitization. Hence, it is also possible that the presence of high concentrations of IgE during phytochemical treatment stabilizes FcεRI expression and thus, we do not see an effect on SYK phosphorylation (Figure 4A and Figure 6A) or BMMC degranulation (Figure 4C). Certainly, IgE has been shown to stabilize FcεRI on the cell surface [62] and increase its localization to the cell membrane over time [42,63]. For TNF production, though upstream signaling pathways do not seem to be inhibited after FcεRI activation, it is also possible that longer term changes to the expression and activation of signaling pathway components downstream of SYK and ERK were still sufficient to affect TNF production. Quercetin has previously been found to affect the expression of several genes involved in various signaling pathways [64]. As the amount of signaling pathways affected is extensive, the identification of the pathways involved in TNF production will be the subject of future research. Notably, a previous study in RBL-2H3 cells found that 10 μm of quercetin was not sufficient to inhibit SYK or ERK phosphorylation [54], possibly explaining why no effect was seen on phosphorylation of these kinases in BMMCs where this concentration was used. Notably, resveratrol was able to affect ERK phosphorylation without affecting the phosphorylation of the upstream kinase SYK. This could potentially be due to resveratrol acting downstream of the phosphorylation event detected by our phospho-SYK antibody. The phospho-SYK antibody we used is directed towards tyrosine 348 (342 in mice/rats) and this tyrosine can be directly phosphorylated by the upstream kinase Lyn [65,66] (Figure 7). If resveratrol directly inhibits the kinase activity of SYK, changes to phosphorylation of this residue may not be affected. Surprisingly, resveratrol at the highest concentration (10 µM) slightly but significantly increased TNF production by BMMC (Figure 4D), but resveratrol did not increase FcεRI expression or potentiate SYK phosphorylation, thus suggesting that these processes are not coupled and dependent upon one another and that some other signaling pathways are responsible for these changes in TNF production.

## 4. Materials and Methods

### 4.1. Cell Culture

MC/9 were cultured in RPMI media (Fisher, Hampton, NH, USA) supplemented with 4 mM L-glutamine (Fisher), 50 μM BME (Sigma-Aldrich, Oakville, ON, Canada), 1 mM sodium pyruvate (Fisher), 100 U/mL penicillin/100 μg/mL streptomycin (Fisher), 0.1 mM nonessential amino acids (Fisher), 25 mM HEPES (Fisher), 10% FBS (Gibco, Burlington, ON, Canada) and 30 ng/mL mouse recombinant interleukin (IL)-3 (Peprotech, Rocky Hill, NJ, USA), pH-7.4–7.6, in a humidified atmosphere of 5% CO_2_ in air at 37 °C.

Femurs were removed from 12-week-old wild type C57Bl strain mice. All animal studies were conducted in accordance with the Canadian Council on Animal Care Guidelines and Policies (https://ccac.ca/en/about-the-ccac/ accessed on 11 May 2022) with approval from the Health Science Animal Care and Use Committee for the University of Alberta. All mouse tissues were a kind gift from Dr. Troy Baldwin, University of Alberta. Bone marrow was aspirated using a 27 gauge needle and the cells were cultured in RPMI media (Fisher, Hampton, NH, USA) supplemented with 4 mM L-glutamine (Fisher), 50 μM BME (Sigma-Aldrich, Oakville, ON, Canada), 1 mM sodium pyruvate (Fisher), 100 U/mL penicillin/100 μg/mL streptomycin (Fisher), 0.1 mM nonessential amino acids (Fisher), 25 mM HEPES (Fisher), 10% FBS (Gibco, Burlington, ON, Canada) and 30 ng/mL mouse recombinant interleukin (IL)-3 (Peprotech, Rocky Hill, NJ, USA), pH-7.4–7.6, in a humidified atmosphere of 5% CO_2_ in air at 37 °C. This media will be referred to as “supplemented RPMI”. The cell suspensions were maintained at a density of 10^5^ cells/mL for 4 weeks when the cells were tested for FcεRI and c-Kit expression by flow cytometry to confirm maturation. After 4 weeks, 99% of cells were double positive for c-Kit and FcεRI. BMMC were used between 4 and 8 weeks of age.

LAD2 MC [67] were cultured in in serum-free StemPro-34 SFM medium (Thermo Fisher Scientific, Waltham, MA, USA) supplemented with 2 mM L-glutamine, 100 U/mL penicillin, 50 µg/mL streptomycin and 100 ng/mL stem cell factor (SCF) (Peprotech Inc., Rocky Hill, CT, USA). The cell suspensions were seeded at a density of 10^5^ cells/mL and maintained at 37 °C and 5% CO_2_. Cells were fed by hemi-depletion of medium once per week. Unless otherwise stated, experiments were performed in StemPro-34 SFM complete with 10 ng/mL SCF.

### 4.2. Trypan Blue Exclusion Assay

MC/9, BMMC and LAD2 were seeded at a density of 2.5 × 10^5^ in a 24-well plate and treated with 1 to 100 µM quercetin, resveratrol or DMSO (0.02%) for 24 h in a humidified chamber at 5% CO_2_ and 37 °C. The cells were mixed with trypan blue (Gibco) at a 1:1 ratio and the percent of viable cells was calculated relative to untreated.

### 4.3. Degranulation Assay

LAD2 were treated with 50 µM resveratrol, quercetin or an equal volume of vehicle control (0.5% DMSO) for 24 h followed by sensitization with 100 ng/mL of human IgE (clone HE1, Invitrogen, Waltham, MA, USA) for 6 h or more. Cells were washed and resuspended in 10 mM HEPES buffer and transferred to a 96 well plate at a concentration of 2.5 × 10^4^ cells/well. Degranulation was induced with 10 µg/mL of anti-Human IgE (Invitrogen) for 30 min before measuring β-hexosaminidase (β-hex) release. The β-hex released into the supernatants and in cell lysates was quantified by hydrolysis of p-nitrophenyl N-acetyl-β-d-glucosamide (Sigma-Aldrich) in 0.1 M sodium citrate buffer (pH 4.5) for 90 min at 37 °C. The percentage of β-hex release was calculated as a percent of total content.

BMMC were seeded at 1 × 10^6^ cell/mL in a 24-well plate and sensitized with 0.5 µg/mL IgE (SPE-7; Sigma-Aldrich) and simultaneously treated with 0.1, 1 or 10 µM quercetin or resveratrol for 24 hr. BMMC were then washed and resuspended in 10 mM HEPES buffer and stimulated with 10 ng/mL DNP-BSA (Invitrogen) in a 96-well plate for 90 min. The β-hexosaminidase released into the supernatants and in cell lysates was quantified by hydrolysis of p-nitrophenyl N-acetyl-β-D-glucosamide (Sigma-Aldrich) in 0.1 M sodium citrate buffer (pH 4.5) for 60 min at 37 °C. The percentage of β-hexosaminidase release was calculated as a percent of total content.

### 4.4. ELISA

MC/9 were seeded at a density of 1 × 10^6^ cells/mL in a 24-well plate and treated with resveratrol or quercetin for 1 h or 24 h in a humidified chamber at 5% CO_2_ and 37 °C followed by sensitization with 0.5 µg/mL IgE (SPE-7, Sigma-Aldrich) for 18 h and stimulation with 100 ng/mL DNP-HA (Invitrogen) for 24 hr. Supernatants were collected and used to assess *de novo* synthesis of TNF. The ELISAs were performed according to manufacturer’s instruction (Applied Biosystems, Waltham, MA, USA) and data was quantified using a standard curve.

BMMC were seeded at a density of 1 × 10^6^ cells/mL in a 24-well plate, sensitized with 0.5 µg/mL IgE (SPE-7; Sigma-Aldrich) and treated with 10 µM of resveratrol or quercetin for 24 h in a humidified chamber at 5% CO_2_ and 37 °C followed by stimulation with 10 ng/mL DNP-BSA (Invitrogen) for 24 h. Supernatants were collected and used to assess *de novo* synthesis of TNF. The ELISAs were performed according to manufacturer’s instruction (Applied Biosystems) and data was quantified using a standard curve.

### 4.5. Flow Cytometric Analysis

MC/9 and BMMC were suspended in supplemented RPMI at a density of 1 × 10^6^ cells/mL so that 0.1 × 10^6^ cells were treated with 1 or 10 µM quercetin, resveratrol or DMSO control for 24 h in a humidified chamber with 5% CO_2_ and 37 °C. Cells were washed 3X in phosphate-buffered saline (PBS) supplemented with 0.5% bovine serum albumin (BSA) (PBS-BSA, Calbiochem Omnipur BSA fraction V) and incubated with 0.006 µg/mL CD117 (c-Kit) PE (eBioscience, San Diego, CA, USA) and 0.006 μg/mL FcεRIα APC (eBioscience) antibodies for 1 h at 4 °C in the dark. Post-staining, cells were washed thrice with PBS-BSA and were re-suspended in 70 μL PBS containing 0.5% BSA/0.05% sodium azide. Samples were analyzed on a CytoFlex flow cytometer (Beckman Coulter, Indianapolis, IN, USA) by acquiring 20,000 events. Rat IgG2b κ PE (eBioscience) and Armenian Hamster IgG APC (eBioscience) were used as isotype controls. Data was analyzed using Flowjo 10.6.2 software.

LAD2 (0.1 × 10^6^ cells) were treated with 50 or 100 µM quercetin, resveratrol or DMSO control for 24 h at 37 °C in a 5% CO_2_ humidified incubator. Sample processing and data analysis for flow cytometry was performed as described above. For detection of human FcεRI and KIT, anti-human FcεRI-APC (eBioscience) and anti-human CD117-PE YB5.B8 (eBioscience) antibodies were utilized. Mouse IgG2b κ-PE (eBioscience) and mouse IgG1 κ-PE (eBioscience) were utilized as the corresponding isotype controls.

### 4.6. Western Blot

LAD2 cells were treated with 50 μM resveratrol, quercetin, an equal volume of vehicle control (0.5% DMSO), or media alone and 100 ng/mL of human IgE (clone HE1, Invitrogen) simultaneously. BMMC were treated with 10 μM resveratrol, quercetin, an equal volume of vehicle control (0.1% DMSO) and 500 ng/mL anti-DNP IgE (Clone Spe7- Sigma-Aldrich). Cells were washed and resuspended in 10 mM HEPES buffer to a concentration of 1 × 10^6^ cells/mL and degranulation was induced with 10 μg/mL of anti-Human IgE (Invitrogen) for 5, 15 or 30 min before lysis in SDS lysis buffer (2% SDS, 100 mM Tris, 10% glycerol, 0.004% bromophenol blue, Halt Phosphatase Inhibitor (Thermo Scientific, Waltham, MA, USA) and cOmplete™, EDTA-free Protease Inhibitor Cocktail (Roche, Basel, Switzerland) and Benzonase (Millipore Sigma). After boiling, a volume of lysate corresponding to 0.5-1x10^6^ cells was loaded on 10% acrylamide gels and SDS-PAGE was conducted, followed by transferring the proteins to a nitrocellulose membrane. Membranes were probed with antibodies directed towards phospho SYK (Y348) (Abcam, Cambridge, UK) and total SYK (Abcam), diphosphorylated ERK1/2 (Sigma-Aldrich) and total ERK1/2 (Cell Signaling, Danvers, MA, USA).

### 4.7. RNA Extraction and cDNA Synthesis

4 × 10^6^ LAD2 were treated with 100 µM resveratrol or quercetin for 3 h and RNA was extracted using the PureLink RNA Mini Kit (Invitrogen, Cat# 12183018A) that employed the on-column DNase (Invitrogen, Cat# 12185010) digestion. The purity and concentration of the RNA was determined using the Nanodrop One (Thermo Scientific) and cDNA was synthesized using 1000 ng of total RNA utilizing the High capacity cDNA reverse transcription kit (Applied Biosystems, cat# 4368814).

### 4.8. qPCR

qPCR was performed utilizing Fast SYBR Green master mix (Applied Biosystems, cat# 438612), human gene specific IDT oligonucleotide primers as described in Table 1 and a StepOnePlus real time PCR machine (Applied Biosystems).

### 4.9. Statistical Analysis

Each experiment was conducted at least three independent times and values displayed represent mean ± standard error of the mean. *p* values were determined by Student’s *t* test (between groups) or one-way ANOVA (comparing more than two groups). A Dunnett’s test was also performed [31], to further validate our statistical analyses.

## 5. Conclusions

Our results show that quercetin-mediated inhibition of MC responses is dependent on the MC model utilized. In mouse MC, quercetin inhibition of MC mediator release is associated with a reduction in the expression of FcεRI and a decrease in TNF production, whereas in the human LAD2, quercetin inhibits FcεRI expression, but has no significant effect on FcεRI signaling or degranulation. Collectively, these results suggest that studies done in rodents may not be extrapolated to humans and that there are likely other pathways, downstream of FcεRI, that are modified by these compounds in both species.

## Figures and Tables

**Table 1:** List of intron spanning human oligonucleotide primers used in this study.

Gene	Forward Primer	Reverse Primer
FcεRI α subunit	gttagcagtccctcagaaacc	ctgccattgtggaaccatttg
FcεRI β subunit FcεRI γ subunit Glyceraldegyde-3-phosphate dehydrogenase (GapDH)	aaatcttgctctcccacagg actgaagatccaagtgcgaaag agccacatcgctcagacac	ggatgaggccgacttcaatag agtctcgtaagtctcctggttc gcccaatacgaccaaatcc

## Data Availability

The datasets supporting the conclusions of this article are included within the article and can be provided upon reasonable request.

## Supplementary Materials

The following supporting information can be downloaded at: https://www.mdpi.com/article/10.3390/molecules27196704/s1, Figure S1: Effect of quercetin and resveratrol on MC/9 viability and optimization of TNF release; Figure S2: Effect of resveratrol and quercetin on BMMC viability; Figure S3: Effect of quercetin and resveratrol on LAD2 cell viability and FcεRI α, β and γ subunit mRNA expression; Figure S4: Full blots for Figure 4A; Figure S5: Full blots for Figure 4B; Figure S6: Full blots for Figure 6A; Figure S7: Full blots for Figure 6B.
