# Supplementary material for: Quercetin and Resveratrol Differentially Decrease Expression of the High-Affinity IgE Receptor (FcεRI) by Human and Mouse Mast Cells
Source: Molecules. 2022 Oct 8;27(19):6704. doi: 10.3390/molecules27196704 (PMC9573482; doi:10.3390/molecules27196704)
Supplement: Supplementary file 1 [file molecules-27-06704-s001.zip › molecules-1899662-supplementary.pdf]

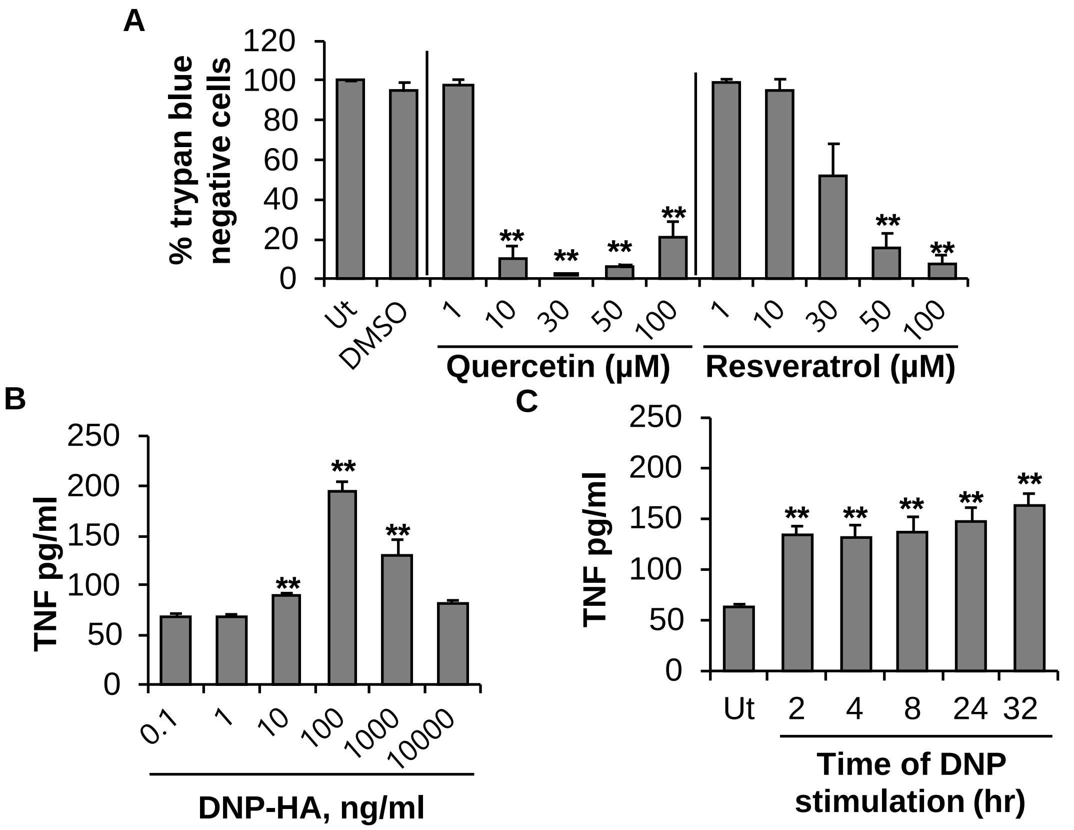

Figure. S1: Effect of quercetin and resveratrol on MC/9 viability and optimization of TNF release. (A) MC/9 were treated with 1, 10, 30, 50 or 100 µM of quercetin, resveratrol or DMSO for 24 hr followed by trypan blue staining. n=3. (B) TNF release by MC/9 following stimulation with different concentrations of DNP-HA (0.1-10,000 ng/ml) for 20 hr at a cell density of $1X10^6$ cells/ml. n=3. (C) TNF release following 100 ng/ml DNP-HA stimulation for different time periods (2-32 hr). Untreated (Ut) represents 32 hr data point. n=3. *p* value <0.01 (**) is relative to 0.1 ng/ml DNP-HA (A, B) or Ut (C).

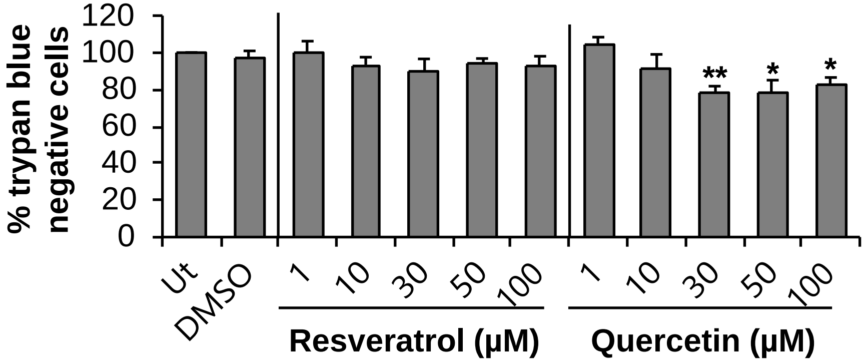

Figure.S2. Effect of resveratrol and quercetin on BMMC viability. BMMC were treated with 1, 10, 30, 50 or 100 µM of the indicated flavonoids or DMSO for 24 hr followed by trypan blue staining. Ut representsuntreated cells. n=5. *p* value <0.05 (*) and <0.01 (**) are relative to DMSO control.

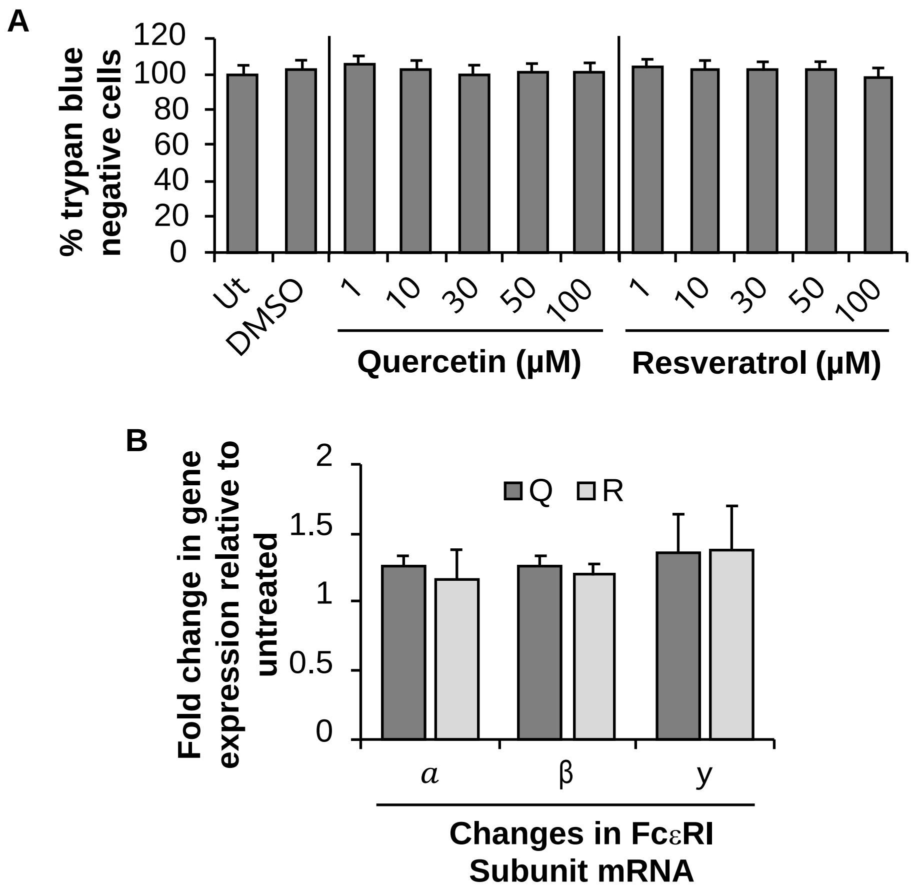

Figure. S3. Effect of quercetin and resveratrol on LAD2 cell viability and FcεRI α, β and γ subunit mRNA expression. (A) LAD2 were treated with 1, 10, 30, 50 or 100 µM of the indicated flavonoids or DMSO for 24hr followed by trypan blue staining. Ut represents untreated cells. n=5. *p* value <0.05 (*) and <0.01 (**) are relative to DMSO control. (B) LAD2 were treated with 100 µM quercetin (Q) or resveratrol (R) for 3 hr followed by RNA extraction, cDNA synthesis and qPCR using gene specific primers against human FcεRI α, β and γ subunits. Human GapDH was utilized as a housekeeping gene to normalize the samples. 2^-!ΔcT value is plotted relative to untreated control. n=3.

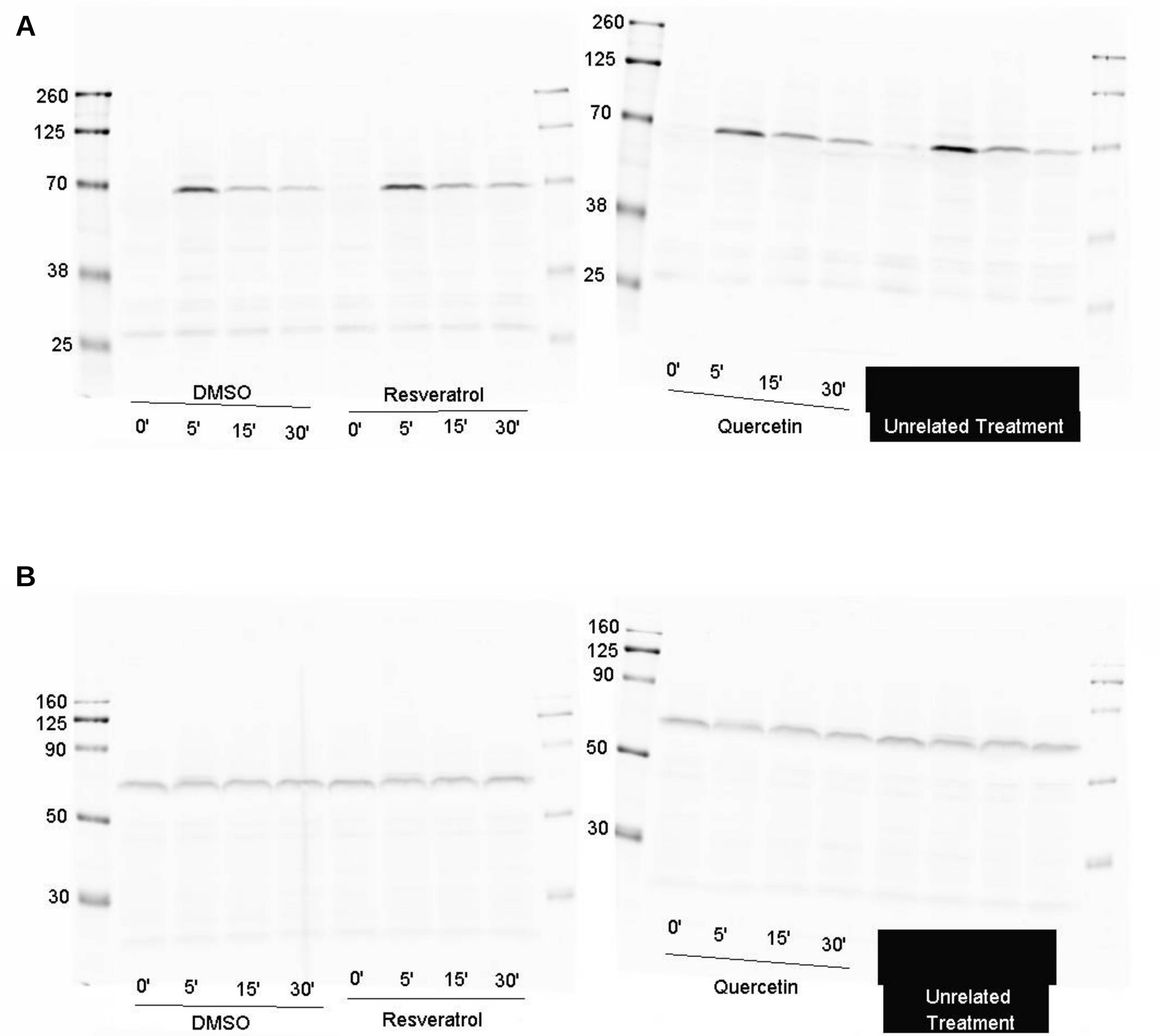

Figure. S4. Full blots for figures 4A. Full western blot images forphospho-SYK (A), and total SYK (B) from figure 4A. Numbers beside ladders indicate predicted size of each MW marker in kDa.

A

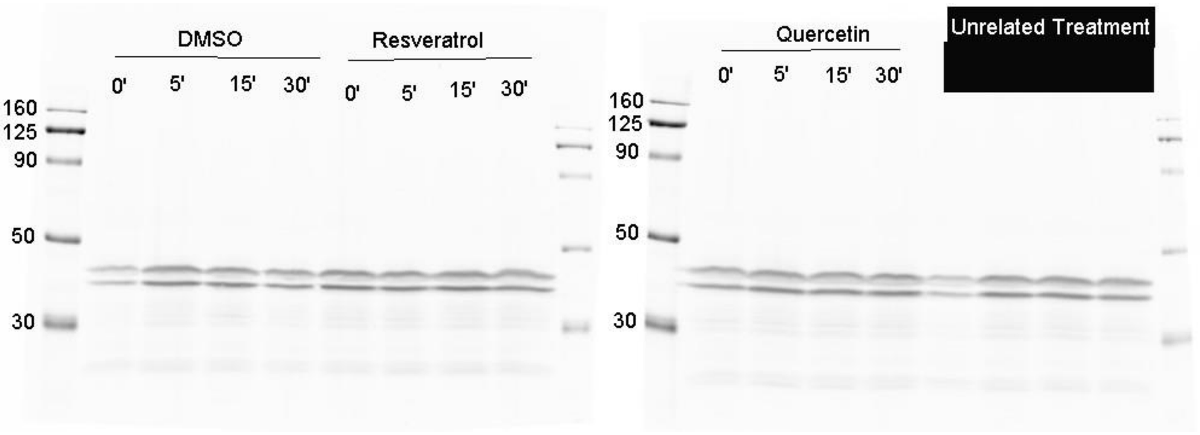

B

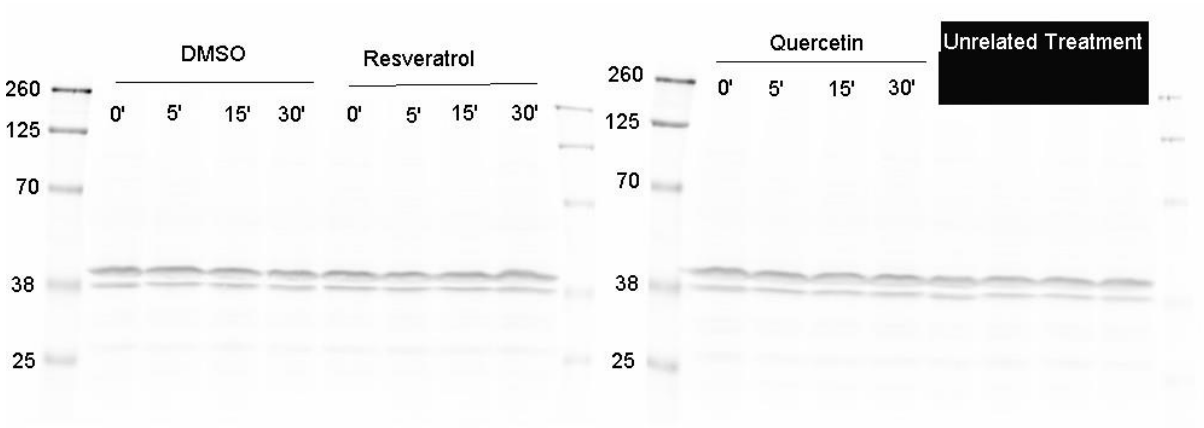

Figure. S5. Full blots for figures 4B. Full western blot images forphospho-ERK (A), and total ERK (B) from figure 4B. Numbers beside ladders indicate predicted size of each MW marker in kDa.

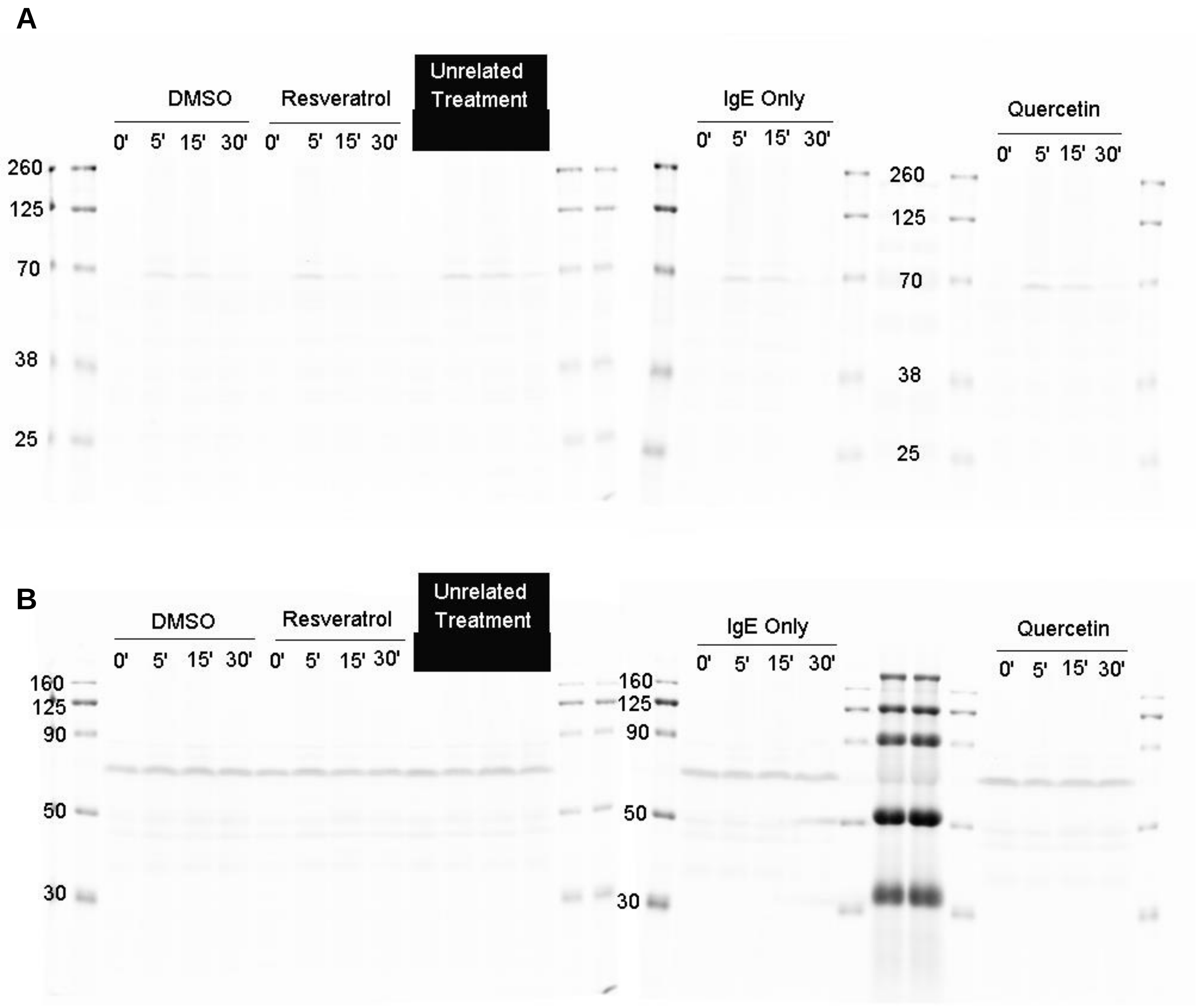

Figure. S6. Full blots for figures 6A. Full western blot images for phospho-SYK (A), total SYK (B) from figure 6A. Numbers beside ladders indicatepredicted size of each MW marker in kDa.

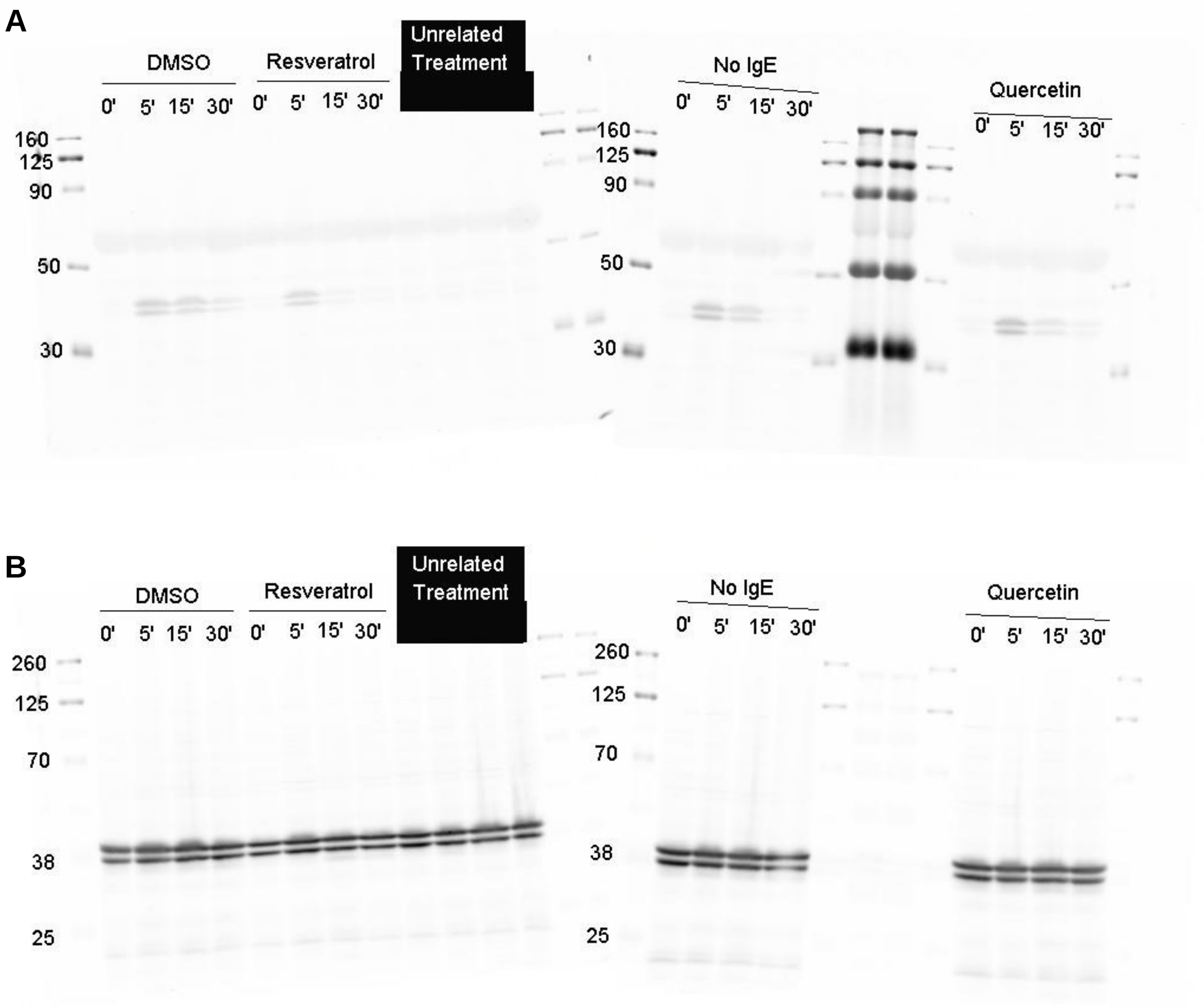

Figure. S7. Full blots for figures 6B. Full western blot images forphospho-ERK (A), and total ERK (B) from figure 6B. Numbers beside ladders indicate predicted size of each MW marker in kDa.
